# Incidental Finding of Nocardia: A Case Series from a Tertiary Care Centre in Uttarakhand

**DOI:** 10.1155/2020/6874625

**Published:** 2020-04-09

**Authors:** Aroop Mohanty, Suneeta Meena, Subodh Pandey Kumar, Puneet Kumar Gupta, Neelam Kaistha, Pratima Gupta, Mithilesh Kumar Jha, Sasi Rekha

**Affiliations:** ^1^Department of Microbiology, All India Institute of Medical Sciences, Rishikesh, Uttarakhand, India; ^2^Department of Laboratory Medicine, All India Institute of Medical Sciences, New Delhi, India; ^3^Department of Emergency Medicine, Rishikesh, Uttarakhand, India

## Abstract

Nocardiosis is a rare bacterial infection that may lead to a severe disease. These infections are rare among normal population and are showing an increasing trend worldwide attributable to the increase in the immunosuppressed population. Most of these patients present with nonspecific clinical features such as fever, productive cough, and exertional dyspnoea as seen in our series of patients which makes it difficult to be diagnosed. Pulmonary nocardiosis is rarely clinically suspected and often diagnosed very late in the course of disease resulting in high mortality. A similar observation was made in one of our cases where the patient was being treated on the lines of pneumonia, and in the end she was diagnosed with pulmonary nocardiosis. In view of the limited literature available, we report here a case series of pulmonary nocardiosis in immunosuppressed patients diagnosed incidentally by fungal KOH mount. The most common conditions causing immunosuppression were type II DM, COPD, and pulmonary tuberculosis.

## 1. Introduction

*Nocardia* are classically soil-borne aerobic, Gram-positive, filamentous, weakly acid-fast bacilli causing opportunistic pulmonary infection in immunocompromised individuals [1]. Airborne droplet is the commonest mode of contracting the infection, making lungs the most affected organ. Pulmonary nocardiosis comprises about 70% of all the total cases of nocardiosis [2]. Globally, the data regarding the incidence of *Nocardia* is very limited. It is rarely clinically suspected and often diagnosed very late in the course of disease [3]. Here, we present case reports of five patients of pulmonary nocardiosis which were incidentally diagnosed after detection of branching filamentous on fungal KOH mount, and one of them was identified as *Nocardia araoensis* by MALDI-TOF MS.

## 2. Case Reports

### 2.1. Case 1

A 64-year-old male patient presented to the pulmonary medicine OPD with complaints of fever and productive cough for five days along with rapidly progressive dyspnoea since the last three days. The fever was high grade in nature and was associated with chills. He also complained of diffuse chest pain and difficulty in passing urine. Five years ago, the patient was diagnosed with chronic obstructive pulmonary disease (COPD) and was also found to be HCV reactive. He had no other significant history. On examination, he was febrile; heart rate was 80 beats/min, respiratory rate was 22/min; blood pressure was 80/60 mm Hg; and oxygen saturation was 88% while breathing room air. Lung auscultation revealed bilateral crepitation scattered throughout both the lung fields. Arterial blood gas analysis revealed type I respiratory failure. On chest radiograph, there was bilateral hyperinflation on both sides. A provisional diagnosis of acute exacerbation of COPD with community-acquired pneumonia was made, and the patient was started on intravenous antibiotics (ceftriaxone and amikacin), high flow oxygen, nebulization with bronchodilators, and intravenous hydrocortisone. Laboratory examination showed leucocytosis (32.3 × 10^3^/*μ*L) and high erythrocyte sedimentation rate (85 mm/h). Sputum sample sent for fungal culture revealed branching filamentous bacilli which were acid fast on modified acid-fast staining (1% H_2_SO_4_) resembling *Nocardia* spp. He was immediately started on Inj Imipenem Cilastatin 500  mg BD. He did not want to further continue his treatment and left against medical advice and was lost to follow-up.

### 2.2. Case 2

A 78-year-old male patient presented to the emergency department with chief complaints of fever with productive cough since last ten days and altered sensorium with chest pain for one day. The patient was apparently well ten days back when he developed high grade fever and cough with haemoptysis and yellowish sputum. It was also associated with shortness of breath. He was a known case of type 2 diabetes mellitus with uncontrolled sugar. There was no past history of taking antitubercular drugs and no exposure to pets. On examination, he was febrile; heart rate was 122 beats/min, respiratory rate was 28/min; blood pressure was 122/75  mm Hg; and oxygen saturation was 84% while breathing room air. The patient had bilateral pedal oedema. Respiratory system examination revealed bilateral crepitation with occasional rhonchi. The total leucocyte count was 14000/mm^3^ with 95% neutrophils and 3% lymphocytes. The haemoglobin was 11.67  g/dl, and the erythrocyte sedimentation rate was 110/h. Serum urea was 43  mg, and creatinine was 0.7  mg/dl. On chest radiography, there were bilateral fluffy opacities. Inj Ceftriaxone 1 gm IV BD was started empirically along with Inj Azithromycin 500  mg BD along with other supportive treatment. On the same day, as the patient was rapidly desaturating, he was shifted to the respiratory ICU where he was intubated and kept on ventilator. Neurologist opinion was taken for the altered sensorium and a normal contrast CT scan of the head was done. Sputum sample was collected which came out negative for AFB but surprisingly KOH mount revealed weak, acid-fast, and filamentous bacilli resembling *Nocardia* spp. On day 4, the empirical regimen was stopped, and the patient was started on Inj Imipenem and Inj Amikacin. However, the patient developed hypotension with tachycardia, as a result of which IV fluids and inotropic support was given. Despite this on day 8, he had severe bradycardia with profound hypotension. CPR was started with repeated cycles and DC shock, but the patient could not be revived and was declared dead.

### 2.3. Case 3

**Figure 1:**
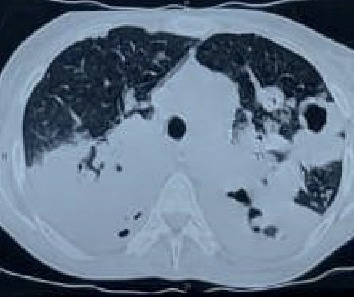
Axial section of CT thorax showing bilateral consolidation with necrosis and cavitation.

**Figure 2:**
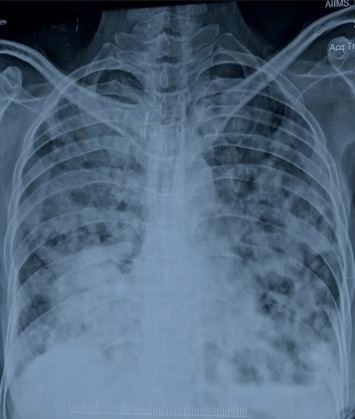
X-ray chest PA view shows bilateral diffuse consolidation and cavitation.

A 47-year-old female patient was referred to our emergency department where she presented with productive cough since 11 months, weight loss for 4 months, shortness of breath, and loss of appetite since last one month. In past history, she was treated as a case of pneumonia with empirical antibiotics at a nearby private teaching hospital where CXR revealed increased opacity. A CECT chest was done which showed left hilar lymphadenopathy with minimal left effusion with focal areas of consolidation (Figure 1). As a result, empirical therapy was changed to broad spectrum antibiotics. She showed relative improvement for a period of 2 months, but then developed weakness of the left upper limb and lower limb with twitching over the face. MRI scan was done at the same private setup which revealed multiple rounded enhancing lesions scattered in the B/L cerebral and cerebellar hemispheres, midbrain, pons, and vermis. A repeat CECT chest done at our hospital showed solid mass in the left hilar region involving the left lower lobe. EBUS (endobronchial ultrasound) guided biopsy from the mediastinal lymph node and endobronchial lesion showed no evidence of malignancy. In view of the high clinical suspicion deterioration of the patient, radiotherapy was given for the space occupying lesions in the brain. As per metastatic workup, a bone scan was performed which showed increased osteoblastic activity in the left 8^th^ and 9^th^ rib anteriorly. A percutaneous lung biopsy was done which showed features suggestive of moderately differentiated squamous cell carcinoma. Chest X-ray was performed immediately after biopsy, and it revealed bilateral diffuse consolidation and cavitation (Figure 2). However, sputum sample sent for KOH mount revealed weak, acid-fast, and filamentous bacilli resembling *Nocardia* spp. She was immediately started on trimethoprim-sulphamethoxazole, linezolid, and imipenem/cilastatin for suspected disseminated nocardiosis. But due to the deterioration of the type II respiratory failure, the condition of the patient worsened, and by the next day, she developed septic shock. She was put on a ventilator but succumbed to her illness despite the best of resuscitative efforts. A final diagnosis of disseminated nocardiosis was made.

### 2.4. Case 4

A 62-year-old female patient presented to the emergency department with chief complaints of breathlessness for 10 years, cough with expectoration, and lower limb swelling since last 4 days. The breathlessness was associated with history of orthopnea, paroxysmal nocturnal dyspnoea (PND), and palpitations. There was a gradual increase in difficulty in breathing over the past 4 days with progression from grade 2 to grade 3. The cough was gradual in onset and was associated with yellowish expectoration and no haemoptysis. The pain in the lower limbs was seen along with abdominal swelling and facial puffiness. The patient was a known case of type 2 DM but was on irregular mediation. There was no history of HTN, PTB, or any other comorbidities. On examination, she was febrile; heart rate was 116 beats/min; respiratory rate was 27 breaths/min; blood pressure was 130/90  mm Hg; and oxygen saturation was 915 while breathing room air. The patient had pallor and bilateral pedal oedema. Systemic examination including respiratory system did not reveal any significant findings. The total leukocyte count was 8520/mm^3^ with 90% neutrophils and 3% lymphocytes. The haemoglobin was 9.6 g/dl, and the erythrocyte sedimentation rate was 102/hr. Serum urea was 75  mg, and creatinine was 2.5  mg/dl. An empirical antibiotic, modified dose of piperacillin and tazobactam was started along with other supportive treatment.

Sputum sample was sent to microbiology laboratory for KOH examination which incidentally revealed weak, acid-fast, and filamentous bacilli resembling *Nocardia* spp. She was immediately initiated on modified dose of trimethoprim/sulfomethaxozole and linezolid. As the patient showed gradual improvement in his condition, he was discharged on the same drugs. He successfully completed 3 months of antibiotics with satisfactory clinico-radiological improvement.

### 2.5. Case 5

A 73-year-old male patient presented to the Pulmonary Medicine OPD with chief complaints of fever, difficulty in breathing, palpitations, and increased frequency of urination since the last 5 days. The fever was low grade in nature and was associated with evening rise of temperature. He also complained of passing blood in urine along with dribbling of the same. He was a known case of type 2 diabetes mellitus and pulmonary tuberculosis since last 10 years and 2 years, respectively. Detailed history revealed that he had taken antitubercular drugs for 6 months and left by himself months back. There was no history of HTN or any other comorbidities. On examination, he was febrile; heart rate was 86 beats/min; respiratory rate was 27 breaths/min; blood pressure was 100/60  mm Hg; and oxygen saturation was 91% while breathing room air. The patient had pallor and bilateral pedal oedema. Respiratory system examination revealed crepitations on the right side of the chest. Other systemic examinations did not reveal any significant findings. The total leukocyte count was 8795/mm^3^ with 70% neutrophils and 18% lymphocytes. His fasting and postprandial blood sugar levels were 185  mg/dl and 439  mg/dl, respectively, whereas HbA1c was recorded as 8.5%. Sputum samples were sent for Gram stain, acid-fast bacilli stain, culture, and KOH mount. AFB stain and GeneXpert was done, both of which came negative. KOH mount revealed branching filament-like structures, which were confirmed by modified acid-fast staining as *Nocardia* spp. The sputum sample was also cultured on blood agar and it revealed white, dry colonies with rough surface after 48 hours of incubation. These colonies were identified by MALDI-TOF as *Nocardia araoensis* with a confidence interval of 1.50. He was started on Tab Trimethoprim-Sulphamethoxazole for a period of 15 days. On follow-up after a period of 15 days, the patient had improved symptomatically.

## 3. Microbiological Diagnosis

**Figure 3:**
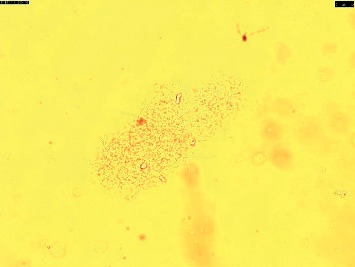
KOH mount showing branching filament-like structures.

**Figure 4:**
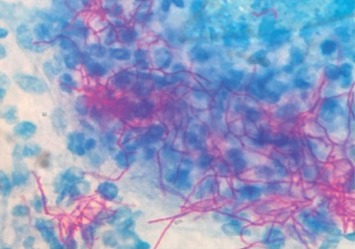
Modified ZN stain showing acid-fast branching filaments.

Sputum sample was sent in all four cases to Microbiology laboratory for acid-fast stain and fungal KOH mount. Acid-fast stain was negative in all four cases, whereas KOH mount incidentally revealed branching filamentous bacteria-like structures (Figure 3). A modified acid-fast staining using 1% sulphuric acid as a decolourizer was made, and it showed weak, acid-fast pink coloured branching filamentous bacilli in all four cases resembling *Nocardia* spp (Figure 4). Out of the five cases mentioned above, growth was obtained in only one case on 5% sheep blood agar, which was successfully identified by MALDI-TOF MS as *Nocardia araoensis* (Table 1) [4].

## 4. Discussion

*Nocardia* spp belong to the aerobic actinomycetes group (phylum: Actinobacteria; order: Actinomycetales) of bacteria. These bacteria can result in human infection either due to inhalation or contact through a cut or abraded skin [5]. Previously, it was thought to be an uncommon infection, but in recent years the incidence of the disease has shown an up rise trend mainly due to increase in the number of immunocompromised individuals due to malignancy, organ transplantation, HIV infection, and chronic glucocorticoid therapy [6, 7]. Among respiratory diseases, COPD is a major risk factor. These patients are most often treated with corticosteroid therapy, and along with their impaired local defences, they become prone to nocardiosis infection more than any other respiratory condition [8, 9]. In our study, diabetes was found to be the most common risk factor for nocardiosis and consisted of 50% of cases followed by COPD and lung malignancy. Three patients also had underlying diabetes. Singh et al. in their study also had similar results and found that diabetes mellitus contributed to 27.8% of the underlying risk factors for nocardiosis.

Lungs are the most common organ to be affected and account for about a majority of all *Nocardia* infections. Pulmonary nocardiosis generally has a subacute (weeks to months) or chronic (months to years) presentation. All our patients had a subacute presentation with a range of 5 days to 11 months prior to presentation. Fever and cough with expectoration were the main symptoms in all our cases (100%). These are usually nonspecific and similar to other bacterial and fungal infections of which pulmonary tuberculosis is a great mimicker [10]. In India, where tuberculosis (TB) is endemic, most of the symptoms are attributed to TB, and the patients are started on anti-TB drugs. Such patients should always be screened for nocardiosis before initiation of anti-TB treatment. In our study, none of the patients had a past history of TB, but one of them was treated on the lines of lung carcinoma which could have been easily prevented by simple early sputum microscopy.

In all the four cases, the microscopic demonstration of branching filament-like structures on KOH mount was an incidental finding. Sputum sample which had been sent to mycology laboratory for presence of fungal elements revealed branching filaments. Modified acid-fast staining using 1% sulphuric acid as a decolorizer was employed to further confirm the presence of *Nocardia*-like species in these samples, where pink-coloured filamentous branching bacilli were observed. It should be noted that isolation of these organisms should always be interpreted with a relevant clinical background, as such bacteria can also be isolated from colonized airways of apparently healthy individuals, such as bronchiectasis [11]. Sample should always be inoculated on blood agar to increase the chances of isolation of *Nocardia*.

Antibiotics were started empirically in all four cases with subsequent modification according to the incidental diagnosis of *Nocardia* infection. Cotrimoxazole is still the drug of choice and is used in combination with other drugs like imipenem-cilastatin, linezolid, and amikacin which have activity against *Nocardia* [12]. A combination of at least two or three intravenous antibiotics were used in all our patients. In this series, one patient due to severe hypotension and the other with lung carcinoma died despite of appropriate medical treatment. Of the other two, one was discharged against medical advice, whereas the second was cured completely. The high mortality rate (50%) as seen here with pulmonary nocardiosis is similar as observed in other studies.

## 5. Conclusion

One should always keep pulmonary nocardiosis as a differential diagnosis from pneumonia for all COPD patients and also in immunocompetent patients with atypical radiological features not responding to empirical treatment. Microbiological isolation of *Nocardia* spp from various clinical specimens is crucial for better patient management in order to prevent the high mortality associated with this condition.

## Figures and Tables

**Table 1:** Clinical and microbiological data of patients.

Age	Sex	Presenting complaints	Underlying disease	KOH	Treatment	Outcome
64	Male	Fever × 5 D	COPD	Positive	Inj Imipenem + Cilastatin	LAMA
		Productive cough × 5 D				
		Dyspnoea × 3 D				

78	Male	Fever × 10 D	Type II DM	Positive	Inj Imipenem–Inj Amikacin	Died
		Productive cough × 10 D				

47	Female	Productive cough × 11 M	Ca lung	Positive	Tab Cotrimoxazole + Inj Linezolid/Inj Imipenem Cilastatin	Died
		Weight loss × 4 M				
		Dyspnoea × 1 M				

62	Female	Dyspnoea × 10 Y	Type II DM	Positive	Tab Cotrimoxazole	Survived
		Productive cough × 4 D				
		Pedal oedema × 4 D				
